# Thyroid Hormone Dysregulation and Lipid Metabolism Alterations in Bipolar Disorder: Associations With Manic Episodes, Aggressive Behaviour and Cognitive Impairment

**DOI:** 10.62641/aep.v53i6.1987

**Published:** 2025-12-17

**Authors:** Juan Guan, Jun Liu, Hong Zhang

**Affiliations:** ^1^Early Intervention Ward 1, Wuhan Mental Health Center, Wuhan Hospital for Psychotherapy, 430000 Wuhan, Hubei, China; ^2^Psychosomatic Ward, Wuhan Mental Health Center, Wuhan Hospital for Psychotherapy, 430000 Wuhan, Hubei, China

**Keywords:** bipolar disorder, thyroid hormones, lipid metabolism, manic, aggressive, cognitive dysfunction, disease severity

## Abstract

**Aims/Background:**

Patients with bipolar disorder experience lipid metabolism disorders and endocrine dysregulation, which may affect emotional regulation, behavioural habits and cognitive function. This study aimed to investigate the correlations amongst lipid metabolism indicators, thyroid hormone levels, and manic episodes, aggressive behaviours, cognitive function and disease severity in patients with bipolar disorder.

**Methods:**

This retrospective analysis included 656 patients with bipolar disorder admitted to Wuhan Mental Health Center. Baseline data, including manic symptoms (Young Mania Rating Scale), aggressive behaviours (Modified Overt Aggression Scale), cognitive function (Montreal Cognitive Assessment), disease severity (Clinical Global Impressions-Severity), lipid metabolism indicators {total cholesterol (TC), triglycerides (TGs), high-density lipoprotein (HDL), low-density lipoprotein (LDL) and thyroid hormone levels [thyroid-stimulating hormone (TSH), total triiodothyronine (T3), total thyroxine (T4), free triiodothyronine (FT3) and free thyroxine (FT4)]}, were collected through electronic medical records.

**Results:**

No statistically significant differences were observed in serum TC, LDL, TSH, T4 or FT3 levels between the manic episode group and the non-manic group (*p* > 0.05). However, the manic episode group exhibited significantly higher serum TG, T3 and FT4 levels (*p* < 0.05) and significantly lower HDL levels (*p* < 0.05) than the non-manic group. No significant differences were observed in serum TC, TG, HDL or LDL levels between the aggressive behaviour group and the non-aggressive group (*p* > 0.05). However, the aggressive behaviour group showed significantly higher TSH, T4, T3, FT3 and FT4 levels (*p* < 0.05) than the non-aggressive group. No significant differences were observed in serum TC, TG, LDL, TSH, T4, T3 or FT3 levels between the cognitive impairment and normal cognition groups (*p* > 0.05). However, the cognitive impairment group had significantly lower HDL and FT4 levels (*p* < 0.05) than the normal cognition group. No statistically significant differences were observed in the serum levels of TC, TG, HDL, LDL, TSH, T4, T3, FT3 or FT4 between the moderate and severe bipolar disorder groups (*p* > 0.05).

**Conclusion:**

Changes in lipid metabolism indicators and thyroid hormone levels in patients with bipolar disorder are closely related to manic episodes, aggressive behaviours and cognitive dysfunction, but no correlation was found with disease severity. This evidence supports precision management of bipolar disorder by utilizing specific lipid and thyroid hormone profiles to guide cardiovascular screening, aggression risk assessment, and early detection of cognitive decline.

## Introduction

Bipolar disorder is a unique and complex mental illness characterised clinically by recurrent episodes of depression and mania. Early symptoms in patients are often nonspecific, and initial onset can occur at any age [1, 2]. While the pathogenesis of bipolar disorder remains incompletely understood, extensive research suggests its development is closely associated with the interplay of biological, psychological and social factors [3, 4, 5]. Manic episodes, as a core clinical phenotype of bipolar disorder, involve neurobiological mechanisms characterised by hyperactivity of the dopaminergic system and dysfunction in the prefrontal–limbic neural circuitry. This state directly determines the selection of acute-phase treatment strategies [6, 7]. Aggressive behaviours exhibit a high prevalence rate amongst patients with bipolar disorder. Their occurrence is closely associated with prefrontal cortical inhibitory deficits and excessive activation of the amygdala [8]. Such behaviours not only cause impairment in patients’ social functioning but also increase emergency department visitation rates and long-term hospitalisation risks [9]. Cognitive dysfunction, serving as a persistent endophenotype in bipolar disorder, involves multidimensional impairments in executive function, working memory and attention, and it may lead to an increased risk of recovery failure [10].

Although significant progress has been achieved in bipolar disorder research in recent years, gaps remain compared with other mental illnesses, particularly regarding the lack of disease-specific biomarkers [11]. Therefore, identifying biomarkers associated with bipolar disorder holds crucial importance for diagnosis, treatment and therapeutic evaluation in affected patients.

Studies have shown that patients with bipolar disorder exhibit lipid metabolism disturbances, which may contribute to the increased prevalence of metabolic syndrome [12, 13]. The underlying mechanisms are proposed to be associated with factors such as emotion-driven dietary behaviour changes and increased steroid hormone levels [14]. Tkachev *et al*. [15] found that alterations in plasma lipids could serve as biomarkers for psychiatric disorders such as bipolar disorder. Through an untargeted lipidomics study on Serbian patients with bipolar disorder, Jadranin *et al*. [16] discovered altered lipid metabolism in their serum. Fusar-Poli *et al*. [17] conducted blood tests on 418 hospitalised patients with bipolar disorder and identified a close correlation between lipid levels and mood episodes. Additionally, metabolomic analysis by Sun *et al*. [18] revealed that glycerolipid metabolism plays a significant role in bipolar disorder. A close association exists between endocrine disorders and mental health [19]. As the largest endocrine organ in the human body, the thyroid gland has been implicated in bidirectional relationships with bipolar disorder development, as evidenced by the following studies: Kuś *et al*. [20] emphasised that even minor changes in thyroid function could modulate the risk of mood disorders such as bipolar disorder; and Spann *et al*. [21] demonstrated that maternal hypothyroxinaemia increases the risk of bipolar disorder in offspring by fivefold. Through Mendelian randomisation analysis, Chen *et al*. [22] revealed that higher free thyroxine (FT4) levels correlate with reduced bipolar disorder risk. Makarow-Gronert *et al*. [23] compared 1122 psychiatric inpatients and identified a significantly higher prevalence of thyroid dysfunction amongst patients with bipolar disorder than those with other psychiatric conditions.

Based on the aforementioned research, lipid metabolism indicators and thyroid hormones are closely associated with bipolar disorder. However, the above-mentioned studies predominantly focus on comparisons between patients with bipolar disorder and healthy populations. Notably, patients with bipolar disorder exhibit impairments in emotional regulation, behavioural habits and cognitive functions. The correlations amongst lipid metabolism indicators, thyroid hormones and these clinical manifestations remain unclear. This study systematically investigated the relationships of lipid metabolism indicators and thyroid hormones with manic episodes, aggressive behaviours, cognitive function and disease severity in patients with bipolar disorder.

## Methods

### Study Participants

This study is a retrospective analysis. A total of 656 patients with bipolar disorder admitted to Wuhan Mental Health Center between January 2016 and January 2023 were selected as study subjects. This study was approved by the Ethics Committee of Wuhan Mental Health Center (KY2024.0813.01). The patients or their guardians signed an informed consent form.

Inclusion criteria:

(a) met the diagnostic criteria for bipolar disorder [24];

(b) moderate to severe disease severity, with a Clinical Global Impressions-Severity (CGI-S) score of 5–7;

(c) educational level of primary school or higher;

(d) able to communicate normally;

(e) no severe physical illnesses; and

(f) complete medical history records.

Exclusion criteria:

(a) comorbid psychiatric disorders;

(b) organic brain injuries;

(c) history of drug or alcohol addiction;

(d) patients with diseases that can cause lipid metabolism and thyroid dysfunction;

(e) severe infectious diseases or haematological disorders; and

(f) pregnant or lactating women.

### Data Collection and Biochemical Indicator Determination

The patients’ baseline data, manic symptoms, aggressive behaviour, cognitive function, disease severity, lipid metabolism indicators and thyroid hormone levels were collected by reviewing electronic medical records.

Baseline data: The baseline data included gender, age distribution, disease course distribution, educational level, marital status, family history of psychiatric disorders (including parents, siblings, grandparents, and maternal grandparents) and body mass index (BMI, weight/height^2^). The BMI classification criteria were adopted from the Guidelines for Prevention and Control of Overweight and Obesity in Chinese Adults (2006) [25].

Mania assessment: Mania was evaluated using the Young Mania Rating Scale [26]. This scale contains 11 items, with four items rated on a 0–8 scale and seven items rated on a 0–4 scale. The total score is the sum of all items. A score of 0–5 indicates no significant manic symptoms (non-manic group), whereas ≥6 indicates presence of manic symptoms (manic group). Bipolar disorder mania has a Cronbach’s α = 0.88–0.92 [27].

Aggressive behaviour: Aggressive behaviour was assessed using the Modified Overt Aggression Scale (MOAS) [28]. The scale contains four subscales (verbal aggression, property aggression, self-aggression and physical aggression towards others) scored between 0 and 4. The total score is calculated as follows: verbal aggression (raw score) + property aggression (×2) + self-aggression (×3) + physical aggression (×4). A total score of ≥4 indicates aggressive behaviour (aggressive group), whereas <4 indicates non-aggressive behaviour (non-aggressive group). Bipolar disorder cohort has a Cronbach’s α = 0.84 [29].

Cognitive function: Cognitive function was evaluated using the Montreal Cognitive Assessment [28]. This 11-item scale covers eight cognitive domains. Participants with ≤12 years of education received +1 adjustment. A total score of ≥26 indicates normal cognition (normal cognition group), whereas <26 indicates cognitive impairment (cognitive impairment group). Bipolar disorder has a Cronbach’s α = 0.762 [30].

Disease severity: Disease severity was assessed using the Clinical Global Impressions-Severity scale (CGI-S) [31]. The scores range from 1 to 7, with 1 = normal, 2 = borderline, 3 = mild, 4 or 5 = moderate (moderate group) and 6 or 7 = severe (severe group). CGI-BP, adapted for bipolar disorder, has a Cronbach’s α = 0.91 [32].

Biochemical indicators: Lipid metabolism indicators [total cholesterol (TC), triglycerides (TGs), high-density lipoprotein (HDL) and low-density lipoprotein (LDL)] were measured using a Roche Cobas 6000 c 501 automated analyser (Roche Diagnostics GmbH, Sandhofer Str. 116, 68305 Mannheim, BW, Germany). Thyroid hormones [thyroid stimulating hormone (TSH), total triiodothyronine (T3), total thyroxine (T4), free triiodothyronine (FT3) and FT4] were measured using ELISA kits (Manufacturer: Shanghai Enzyme-linked Biotechnology Co., Ltd.; Origin: China, Cat#: ml104029, ml105456, ml106408, ml105365 and ml061037, respectively). All serological indicators were collected under standardised fasting conditions in the morning.

### Statistical Analysis

The results were compiled and entered into Excel (2013, Microsoft Corporation, Redmond, WA, USA). Continuous variables were assessed for normality by using *Kolmogorov–Smirnov test*. The serum levels of TC, TG, HDL, LDL, TSH, T4, T3, FT3 and FT4 exhibited non-normal distributions (these variables were subjected to natural logarithm transformation and square-root transformation. All analyses were ultimately performed using the non-parametric module because the transformed data still violated normality requirements). They were expressed as median and quartiles (P25, P75), with statistical analysis performed using *Mann–Whitney U test*. Categorical variables (including baseline data such as gender, age distribution, disease course distribution, educational level, marital status, family history of psychiatric disorders and BMI) were presented as [*n* (%)]. A *p* < 0.05 was considered statistically significant. Statistical analyses were conducted using SPSS (version 23.0) software (International Business Machines Corporation, Armonk, NY, USA).

## Results

### Baseline Characteristics

The baseline characteristics of 656 patients with bipolar disorder are presented in Table 1.

**Table 1.** **Baseline characteristics of 656 patients with bipolar disorder**.

Characteristics		All patients (*n* = 656)
Gender [*n* (%)]		
	Male	318 (48.48)
	Female	338 (51.52)
Age distribution [*n* (%)]		
	18–20 years	121 (18.45)
	21–30 years	310 (47.26)
	31–40 years	127 (19.36)
	41–50 years	61 (9.30)
	51–60 years	37 (5.64)
Disease duration [*n* (%)]		
	≤5 years	313 (47.71)
	6–20 years	303 (46.19)
	>20 years	40 (6.10)
Educational level [*n* (%)]		
	Primary school/Junior high school	139 (21.19)
	Technical school/High school diploma	159 (24.24)
	College/Bachelor’s degree or higher	358 (54.27)
Marital status [*n* (%)]		
	Married	212 (32.32)
	Unmarried	398 (60.67)
	Divorced/Widowed	46 (7.01)
Family history of psychiatric disorders [*n* (%)]		
	Yes	204 (31.10)
	No	452 (68.90)
BMI [*n* (%)]		
	<18.50 kg/m^2^	73 (11.13)
	18.50–24.0 kg/m^2^	364 (55.49)
	≥24.0 kg/m^2^	219 (33.38)
Manic episode [*n* (%)]		
	Yes	138 (21.04)
	No	518 (78.96)
Aggressive behaviour [*n* (%)]		
	Yes	197 (30.03)
	No	459 (69.97)
Cognitive impairment [*n* (%)]		
	Yes	308 (46.95)
	No	348 (53.05)

### Comparison of Lipid Metabolism Indicators and Thyroid Hormone Levels Between Manic Episode Group and Non-Manic Group

No statistically significant differences were observed in serum TC, LDL, TSH, T4 or FT3 levels between the manic episode group and the non-manic group (*p* > 0.05). However, the manic episode group exhibited significantly higher serum TG, T3 and FT4 levels (*p* < 0.05) and significantly lower HDL levels (*p* < 0.05) than the non-manic group, as shown in Table 2.

**Table 2.** **Comparison of lipid metabolism indicators and thyroid hormone levels between manic episode group and non-manic group**.

Factor	Manic episode group (n = 138)	Non-manic group (n = 518)	Z-value	*p*-value
TC (mmol/L)	4.17 (3.70, 4.65)	4.23 (3.62, 4.85)	−0.896	0.370
TG (mmol/L)	1.29 (0.85, 1.88)	1.09 (0.81, 1.59)	−1.980	0.048
HDL (mmol/L)	1.13 (0.98, 1.24)	1.21 (1.02, 1.44)	−4.130	<0.001
LDL (mmol/L)	2.21 (1.80, 2.57)	2.20 (1.73, 2.74)	−0.069	0.945
TSH (µIU/mL)	2.03 (1.33, 2.98)	1.89 (1.23, 3.11)	−0.765	0.444
T4 (µg/dL)	7.60 (6.58, 9.11)	7.72 (6.59, 9.13)	−0.203	0.839
T3 (ng/mL)	0.97 (0.89, 1.21)	0.95 (0.80, 1.09)	−2.940	0.003
FT3 (pg/mL)	2.92 (2.51, 3.29)	2.95 (2.58, 3.33)	−0.665	0.506
FT4 (ng/dL)	1.08 (0.91, 1.31)	1.00 (0.82, 1.20)	−2.478	0.013

Abbreviations: TC, total cholesterol; TG, triglyceride; HDL, high-density lipoprotein; LDL, low-density lipoprotein; TSH, thyroid-stimulating hormone; T4, total thyroxine; T3, total triiodothyronine; FT3, free triiodothyronine; FT4, free tetraiodothyronine.

### Comparison of Lipid Metabolism Indicators and Thyroid Hormone Levels Between Aggressive Behaviour Group and Non-Aggressive Group

No significant differences were observed in serum TC, TG, HDL or LDL levels between the aggressive behaviour group and the non-aggressive group (*p* > 0.05). However, the aggressive behaviour group showed significantly higher TSH, T4, T3, FT3 and FT4 levels (*p* < 0.05) than the non-aggressive group, as detailed in Table 3.

**Table 3.** **Comparison of lipid metabolism indicators and thyroid hormone levels between aggressive behaviour group and non-aggressive group**.

Factor	Aggressive behaviour group (n = 197)	Non-aggressive group (n = 459)	Z-value	*p*-value
TC (mmol/L)	4.22 (3.58, 4.74)	4.17 (3.66, 4.82)	−0.621	0.534
TG (mmol/L)	1.03 (0.79, 1.73)	1.11 (0.83, 1.66)	−0.789	0.430
HDL (mmol/L)	1.15 (0.98, 1.39)	1.21 (1.02, 1.41)	−1.115	0.265
LDL (mmol/L)	2.21 (1.68, 2.67)	2.20 (1.76, 2.70)	−0.574	0.566
TSH (µIU/mL)	2.18 (1.33, 4.61)	1.88 (1.20, 2.92)	−2.798	0.005
T4 (µg/dL)	8.47 (7.19, 10.05)	7.50 (6.38, 8.82)	−5.588	<0.001
T3 (ng/mL)	1.18 (0.90, 1.28)	0.93 (0.79, 1.02)	−9.224	<0.001
FT3 (pg/mL)	3.28 (2.73, 3.72)	2.87 (2.51, 3.20)	−6.994	<0.001
FT4 (ng/dL)	1.14 (0.92, 1.47)	0.99 (0.80, 1.15)	−6.912	<0.001

Abbreviations: TC, total cholesterol; TG, triglyceride; HDL, high-density lipoprotein; LDL, low-density lipoprotein; TSH, thyroid-stimulating hormone; T4, total thyroxine; T3, total triiodothyronine; FT3, free triiodothyronine; FT4, free tetraiodothyronine.

### Comparison of Lipid Metabolism Indicators and Thyroid Hormone Levels Between Cognitive Impairment Group and Normal Cognition Group

No significant differences were observed in serum TC, TG, LDL, TSH, T4, T3 or FT3 levels between the cognitive impairment and normal cognition groups (*p* > 0.05). However, the cognitive impairment group had significantly lower HDL and FT4 levels (*p* < 0.05) than the normal cognition group, as shown in Table 4.

**Table 4.** **Comparison of lipid metabolism indicators and thyroid hormone levels between cognitive impairment group and normal cognition group**.

Factor	Cognitive impairment group (n = 308)	Normal cognition group (n = 348)	Z-value	*p*-value
TC (mmol/L)	4.17 (3.57, 4.68)	4.26 (3.66, 4.87)	−1.243	0.214
TG (mmol/L)	1.09 (0.84, 1.49)	1.10 (0.80, 1.93)	−1.270	0.204
HDL (mmol/L)	1.11 (0.98, 1.22)	1.32 (1.05, 1.51)	−8.173	<0.001
LDL (mmol/L)	2.27 (1.82, 2.68)	2.15 (1.67, 2.70)	−1.835	0.066
TSH (µIU/mL)	1.89 (1.23, 2.99)	1.96 (1.25, 3.16)	−0.597	0.551
T4 (µg/dL)	7.51 (6.32, 9.03)	7.86 (6.81, 9.17)	−1.913	0.056
T3 (ng/mL)	0.95 (0.80, 1.12)	0.97 (0.83, 1.10)	−0.576	0.565
FT3 (pg/mL)	2.94 (2.53, 3.33)	2.94 (2.59, 3.33)	−0.723	0.470
FT4 (ng/dL)	1.01 (0.81, 1.17)	1.02 (0.84, 1.33)	−2.621	0.009

Abbreviations: TC, total cholesterol; TG, triglyceride; HDL, high-density lipoprotein; LDL, low-density lipoprotein; TSH, thyroid-stimulating hormone; T4, total thyroxine; T3, total triiodothyronine; FT3, free triiodothyronine; FT4, free tetraiodothyronine.

### Comparison of Lipid Metabolism Indicators and Thyroid Hormone Levels Between Moderate and Severe Bipolar Disorder Groups

No statistically significant differences were observed in the serum levels of TC, TG, HDL, LDL, TSH, T4, T3, FT3 or FT4 between the moderate and severe bipolar disorder groups (*p* > 0.05), as shown in Table 5.

**Table 5.** **Comparison of lipid metabolism indicators and thyroid hormone levels between moderate and severe bipolar disorder groups**.

Factor	Moderate bipolar disorder group (n = 371)	Severe bipolar disorder group (n = 285)	Z-value	*p*-value
TC (mmol/L)	4.25 (3.61, 4.89)	4.14 (3.66, 4.73)	−0.613	0.540
TG (mmol/L)	1.10 (0.81, 1.66)	1.09 (0.81, 1.68)	−0.028	0.977
HDL (mmol/L)	1.18 (1.00, 1.39)	1.20 (1.01, 1.41)	−0.562	0.574
LDL (mmol/L)	2.22 (1.71, 2.77)	2.20 (1.76, 2.61)	−0.682	0.495
TSH (µIU/mL)	1.94 (1.24, 3.00)	1.91 (1.23, 3.16)	−0.060	0.952
T4 (µg/dL)	7.80 (6.68, 9.24)	7.58 (6.40, 9.08)	−1.074	0.283
T3 (ng/mL)	0.97 (0.82, 1.11)	0.95 (0.82, 1.10)	−0.642	0.521
FT3 (pg/mL)	2.95 (2.58, 3.32)	2.93 (2.55, 3.36)	−0.140	0.889
FT4 (ng/dL)	1.02 (0.84, 1.23)	1.01 (0.82, 1.23)	−0.736	0.462

Abbreviations: TC, total cholesterol; TG, triglyceride; HDL, high-density lipoprotein; LDL, low-density lipoprotein; TSH, thyroid-stimulating hormone; T4, total thyroxine; T3, total triiodothyronine; FT3, free triiodothyronine; FT4, free tetraiodothyronine.

## Discussion

In this study, the clinical data of 656 patients with bipolar disorder were retrospectively analysed. The findings revealed that 138 patients experienced manic episodes, 197 exhibited aggressive behaviours, 308 presented with cognitive dysfunction and disease severity was categorised as moderate in 371 cases and severe in 285 cases. Additionally, the study demonstrated that the manic episode group had significantly higher serum levels of TG, T3 and FT4 than the non-manic episode group, whereas their HDL levels were significantly lower. The aggressive behaviour group showed significantly increased serum levels of TSH, T4, T3, FT3 and FT4 than patients without aggressive behaviours. Furthermore, patients with cognitive dysfunction exhibited significantly lower serum HDL and FT4 levels than the cognitively normal group.

Manic episodes are a clinical manifestation of bipolar disorder, characterised primarily by increased mood, racing thoughts and increased goal-directed activities. The findings of this study suggest associations between manic episodes and the levels of TG and HDL. Liu *et al*. [33] found a higher risk of manic episodes in spring amongst patients with bipolar disorder and proposed a potential link to seasonal variations in serum TG levels. Li *et al*. [34] investigated outpatients with bipolar disorder and reported a higher prevalence of hyperuricemia in those experiencing manic episodes, hypothesising a connection with increased serum TG levels. Sırlıer Emir *et al*. [35] observed lower HDL values and increased criminal risk in patients with bipolar disorder diagnosed with manic episodes. Whilst these studies indirectly support the relevance of TG and HDL to manic episodes in bipolar disorder, the present research directly confirms this correlation, consistent with the conclusions of Shapiro *et al*. [36]. Regarding thyroid hormone levels, the present study identified higher serum T3 and FT4 levels in the manic episode group. Notably, existing literature has not reported similar findings, except for Zhao *et al*. [37], who documented increased FT3 levels in patients with manic bipolar disorder—a result diverging from the conclusions of the present study. The underlying mechanisms remain unreported in current literature. This phenomenon may be related to a thyroid hormone-neuroinflammation positive feedback loop, wherein inflammatory cytokines during manic states further compromise blood–brain barrier integrity.

Aggressive behaviour is a common adverse behaviour amongst patients with mental disorders. Early research [38] indicated a potential association between thyroid dysfunction and psychiatric symptoms such as mood changes and irritability. Eklund *et al*. [39] found increased serum T3 levels in males with criminal histories, suggesting that higher T3 levels correlate with increased risks of antisocial behaviour and violence. Turgay [40] proposed that hyperthyroidism may be linked to aggressive behaviour in psychiatric patients. Sinai *et al*. [41], in a study of women with borderline personality disorder, identified a significant positive correlation between serum T3 levels and interpersonal violence in adulthood. Acar and Ulgen [42] further noted associations between increased T3/T4 levels and aggressive, violent criminal behaviours. The present study revealed significantly increased thyroid hormone levels across all measured parameters in patients with aggressive behaviours. To the authors’ knowledge, this study is the first to demonstrate such findings, specifically in patients with bipolar disorder. The underlying mechanism may involve heightened central nervous system excitability induced by excessive thyroid hormone levels [43, 44].

A study has demonstrated widespread social cognitive impairment in patients with bipolar disorder [45]. The present study found that the cognitive dysfunction group exhibited decreased levels of HDL and FT4. Zou *et al*. [46], in a study of female patients with bipolar disorder, reported abnormalities in glucose-lipid and reproductive hormone levels amongst those with comorbid cognitive dysfunction. Hui *et al*. [47] highlighted that cognitive function in patients with bipolar disorder is significantly lower than in healthy controls, proposing that reduced serum HDL levels correlate with cognitive impairment. HDL, a complex lipoprotein primarily composed of apolipoprotein A-I (ApoA-I), demonstrates antioxidant and anti-inflammatory properties [48]. Fitz *et al*. [49] further revealed through animal experiments that ApoA-I is associated with β-amyloid clearance rates, suggesting that reduced HDL levels may impair cognitive function in patients with bipolar disorder by affecting cerebral β-amyloid clearance. Regarding thyroid hormones, Barbero *et al*. [50] observed that higher FT4 levels correlate with better cognitive performance in affective disorders like bipolar disorder, a conclusion supported by Lai *et al*. [51] and consistent with the findings of the present study. The underlying mechanism may involve a metabolic-inflammatory cascade: FT4 deficiency potentially triggers compensatory hyperactivity of the hypothalamic–pituitary–adrenal axis, thus heightening cortisol levels. This hypercortisolaemic state further primes microglial TLR4/NF-κB pathway activation, ultimately inducing neuronal apoptosis. Qiu *et al*. [52] identified associations between cognitive dysfunction and increased serum TG levels in patients with bipolar disorder through glucose-lipid metabolism assessments—a finding divergent from the observation of “no significant TG level differences across cognitive function groups”. This discrepancy may stem from variations in sample size, age, geographic distribution or cognitive assessment methodologies.

This study provides the following critical insights for the clinical management of bipolar disorder: Increased serum TG and reduced HDL levels suggest that dyslipidaemia may contribute to manic pathophysiology, necessitating rigorous cardiovascular risk screening in patients with bipolar disorder. Concurrent increases in T3 and FT4 levels indicate thyroid hormone involvement in manic dysregulation, warranting vigilance for comorbid hyperthyroidism. A pan-increase in TSH and thyroid hormones (T4, T3, FT3 and FT4) implies hyperactive hypothalamic–pituitary–thyroid axis function as a potential aggression amplifier. Clinical recommendations include integrating thyroid function testing into bipolar disorder aggression risk assessment protocols. Decreased HDL levels imply compromised lipid-mediated neurorepair mechanisms, and reduced FT4 levels reflect diminished thyroid support for cognition. Dual monitoring of HDL and FT4 enables early detection of bipolar disorder-associated cognitive decline.

This study has several limitations. Firstly, as a single-center retrospective analysis, this research is constrained by a limited sample size, single-source sampling and difficulty in fully eliminating confounding variables, which restricts the generalisability and validity of the conclusions. Secondly, the medications prescribed to patients with bipolar disorder varied, potentially introducing interference from drugs and their metabolites into the results. Additionally, differences in dietary and exercise patterns may influence physiological and biochemical indicators such as lipid profiles. This study did not account for confounding factors, such as age, sex, BMI and illness duration, in analysing associations between clinical variables and biochemical indicators. This study did not systematically analyse demographic characteristic differences amongst clinical subtypes. In subsequent research, mechanistic exploration could be deepened through prespecified multivariable regression models and causal inference approaches to enhance methodological rigor. The sample size and geographic diversity of participants could be expanded; prospective trials could be conducted; and the effects of medications, diet and exercise could be comprehensively accounted for to enhance the accuracy of findings.

## Conclusion

Alterations in lipid metabolism indicators and thyroid hormone levels are associated with manic episodes, aggressive behaviour and cognitive dysfunction in patients with bipolar disorder, suggesting their potential utility as auxiliary predictive markers for these clinical symptoms. Clinical management should integrate individualised interventions targeting lipid metabolism and thyroid hormone imbalances to optimise therapeutic outcomes.

## Availability of Data and Materials

The data analyzed was available on the request for the corresponding author.
